# Responses of butter leaf lettuce to mixed red and blue light with extended light/dark cycle period

**DOI:** 10.1038/s41598-022-10681-3

**Published:** 2022-04-28

**Authors:** Xiao-li Chen, You-li Li, Li-chun Wang, Qi-chang Yang, Wen-zhong Guo

**Affiliations:** 1 Intelligent Equipment Research Center, Beijing Academy of Agriculture and Forestry Sciences, Beijing, 100097 China; 2 Institute of Environment and Sustainable Development in Agriculture, Chinese Academy of Agricultural Sciences, Beijing, 100081 China; 3 Key Laboratory of Urban Agriculture (North China), Ministry of Agriculture and Rural Affairs, Beijing, China

**Keywords:** Plant sciences, Environmental sciences

## Abstract

To investigate the effects of extended light/dark (L/D) cycle period (relative to the diurnal L/D cycle) on lettuce and explore potential advantages of abnormal L/D cycles, butter leaf lettuce were grown in a plant factory with artificial light (PFAL) and exposed to mixed red (R) and blue (B) LED light with different L/D cycles that were respectively 16 h light/8 h dark (L16/D8, as control), L24/D12, L48/D24, L96/D48 and L120/D60. The results showed that, all the abnormal L/D cycles increased shoot dry weight (DW) of lettuce (by 34–83%) compared with the control, and lettuce DW increased with the L/D cycle period prolonged. The contents of soluble sugar and crude fiber in lettuce showed an overall upward trend with the length of L/D cycle extended, and the highest vitamin C content as well as low nitrate content were both detected in lettuce treated with L120/D60. The light use efficiency (LUE) and electric use efficiency (EUE) of lettuce reached the maximum (respectively 5.37% and 1.76%) under L120/D60 treatment and so were DW, Assimilation rate (A), RC/CS, ABS/CS, TR_o_/CS and DI_o_/CS, indicating that longer L/D cycle period was beneficial for the assimilation efficiency and dry matter accumulation in lettuce leaves. The highest shoot fresh weight (FW) and nitrate content detected in lettuce subjected to L24/D12 may be related to the vigorous growth of root, specific L/D cycle seemed to strengthen root growth and water absorption of lettuce. The openness level of RC in PSII (Ψ_o_), ET_o_/CS, and PI_abs_ were all the highest in lettuce treated with L24/D12, implying that slightly extending the L/D cycle period might promote the energy flowing to the final electron transfer chain. In general, irradiation modes with extended L/D cycle period had the potential to improve energy use efficiency and biomass of lettuce in PFAL. No obvious stress or injury was detected in lettuce subjected to prolonged L/D cycles in terms of plant growth and production. From the perspective of shoot FW, the optimal treatment in this study was L24/D12, while L120/D60 was the recommended treatment as regards of the energy use efficiency and nutritional quality.

## Introduction

Light is not only the energy source of plant photosynthesis, but also environmental signal that controls many aspects of plant growth and development. The light factors that affect plant growth and substance metabolism include light intensity, light quality, photoperiod and light distribution. Due to complicated and dynamic changes in natural light, the regulation on light environment is very limited in cultivation facilities depending on natural light. On the contrary, based on the development of artificial light sources especially light emitting diodes (LEDs), comprehensive and accurate control can be conducted on light environment in the artificial light dependent cultivation facilities. Different light qualities have varied effects on plants, among which, red (R) and blue light (B) corresponding to the maximum absorption spectrum of photosynthetic pigments have the most important impacts on plants. R acts a crucial role in the development of photosynthetic organs, morphogenesis, and the synthesis of photochemical substances such as phenols and oxalic acid^1–4^; B is indispensable in chloroplast development, chlorophyll formation, stomatal opening, morphogenesis and anthocyanin synthesis^5–8^. Studies have shown that plants can not grow normally under pure R or pure B, mixed R and B have been considered more suitable for plant growth^9,10^. LED provides the possibility to precisely control the proportions of R and B in mixed RB due to the single spectrum and adjustable light factors. The responses of plants to R/B ratios in mixed RB are specific to species, as regards of lettuce, the R/B ratio of 9:1 has been reported as efficient light formula for growth and quality^10^.

In artificial light dependent cultivation facilities, lighting supply modes can be divided into two types, that are intermittent irradiation mode and continuous irradiation mode. Light and darkness alternates in regular cycles in intermittent irradiation mode, while there is only light period but no dark period in continuous irradiation mode^11,12^. The light/dark (L/D) cycle is characterized by two factors that are the length of L/D cycle (period) and the ratio of the illumination time to the dark time (L/D ratio). It is called normal L/D cycle when the L/D cycle period is equal to the diurnal cycle period 24 h (h), otherwise it is called abnormal L/D cycle^13–15^. So far, related studies have mainly focused on light intensity and light quality, however, only a few reports have studied extensively about the influence of intermittent light irradiation on plant production.

According to some previous studies, the abnormal L/D cycle generated by artificial light might enhance the productivity of valuable secondary metabolites by plant tissue. For example, Kurata^16^ conducted intermittent irradiation with the L/D cycles of 6 h /6 h, 12 h/12 h, 24 h/24 h, and 36 h/36 h on coffea arabica cell, finding that cell growth was enhanced with the L/D cycle period increased despite the same amount of light illumination (intensity × time). Kurata^17^ also investigated the L/D cycle with second-scale periods, proposing that intermittent irradiation with L/D cycle of 2S/18S (s: second) resulted in the same level of caffeine production in coffea arabica cell as the continuous light irradiation. This indicated that the shortened L/D cycle increased the production efficiency regarding light consumption by 10 times compared with continuous light irradiation. Moreover, Kurata^18^ pointed that intermittent illumination with a second-scale period produced the same amount of anthocyanin in strawberry cell as continuous light, while the hour-scale cycle operation decreased anthocyanin production in strawberry cell. This suggested that the alternating interval of light and dark had important influence on the cultural effects of plant tissue. There have also been some studies available about intermittent irradiation on wheat and potato. Dong^19^ reported that intermittent lighting modes such as L/D (0.5 ms/0.5 ms), L/D (0.7 ms/0.3 ms) and L/D (0.8 ms/0.2 ms) increased photosynthetic rate of wheat plants compared with the continuous light. Sivakumar^20^ found that the dry weight and carbohydrate content in sweet potato seedlings under intermittent light with a certain L/D cycle were higher than those under continuous light. As mainly used light spectrum in PFAL, R and B have been mostly concerned in terms of the light intensity and R/B ratio, while the LUE or EUE of plants under different lighting modes of RB have rarely been concerned. Few studies or applications involving unconventional L/D cycles with mixed RB have been reported, and the impacts of different L/D cycles with mixed RB on lettuce is unclear.

Although the application of light-emitting diodes (LEDs) in horticulture keeps expanding owing to the characteristics such as controllable spectral composition, linear photon output and cool emitting surface^21,22^, the cost of LEDs and the energy consumption of LED lighting source are still key factors restricting their large-scale applications. Energy efficiency and photosynthetic efficiency are important indicators determining the operation cost of artificial light source in PFAL. In our previous study, we found that abnormal L/D cycle i.e. 8 h/4 h not only significantly increased shoot DW but also improved the flavor of lettuce compared with the conventional L/D cycle i.e. 16 h/8 h based on the same total light amount^23^. This showed that intermittent light with different L/D cycles had the potential to enhance energy use efficiency by optimizing yield or other targeted traits. Furthermore, if L/D cycle period is extended, the LED light source may be used alternately among the cultivation areas, thereby improving the utilization rate of the LED light source. Therefore, intermittent RB irradiation with extended L/D cycle period was conducted on lettuce in the present study to analyze the effects of unconventional L/D cycles on lettuce from the perspectives of energy utilization, photosynthetic efficiency and nutrient quality of lettuce. Moreover, the study also aimed to investigate the possibility of enhancing the use efficiency of both electric energy and LED light source in plant factory via extending the L/D cycle period.

## Methodology

### Experimental set-up and growth conditions

The experiment was conducted in a plant factory of BAAFS, Beijing, China. In the plant factory, the temperature, air humidity and the CO_2_ concentration were respectively set at 24 °C/20 °C (day/night), 65% and 450 μmol·mol^−1^. Butter leaf lettuce (*Lactuca sativa* L. ‘Flandria’; Rijk Zwaan Co., Netherlands) were hydroponically cultured in sponges for 14 days under white LED light (5500 k, Ra90) at 100 μmol·m^−2^·s^−1^ PPFD (photosynthetic photon flux density) and then the seedlings were transferred into hydroponic boxes for different lighting treatments. The pH and EC of Hoagland’s nutrient solution were about 6.5 and 1.30 mS·cm^−1^ respectively, and the nutrient solution was renewed every 10 days. Harvest was carried out on the 44th day after sowing (i.e. 30 days after planting). Five plants randomly taken from per treatment was regarded as a repetition, and there were three repetitions in each treatment.

**Figure 1:**
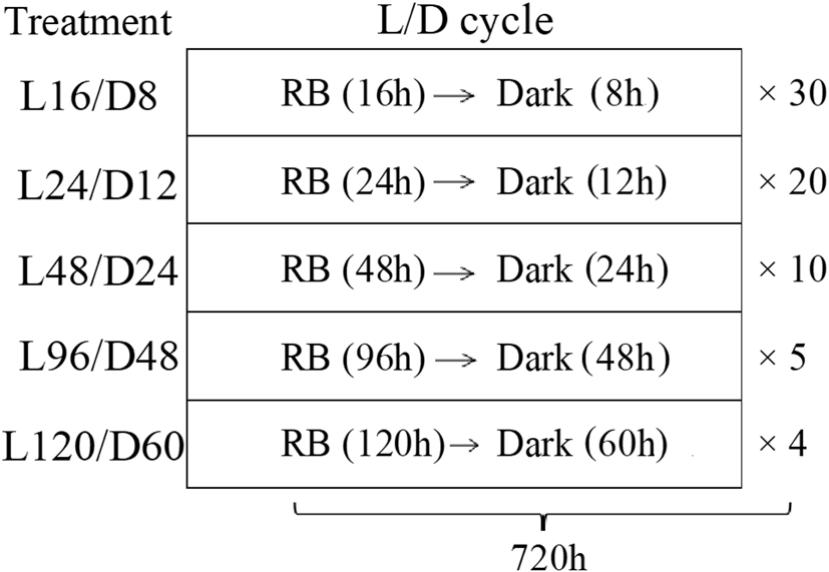
The irradiation modes of RB (mixed red and blue LED light, R:B = 9:1) over a 720-h growth period in different treatments. L/D—light/dark. L16/D8—16 h light/8 h dark (as control), L24/D12—24 h light/12 h dark, L48/D24—48 h light/24 h dark, L96/D48—96 h light/48 h dark, L120/D60—120 h light/60 h dark, there were respectively 30, 20, 10, 5 and 4 L/D cycles in the growth period in each treatment.

Light factors in the study were regulated precisely by LED control system developed by BAAFS. All tested plants were subjected to mixed R (peak at 660 nm) and B (peak at 450 nm) with total PPFD of 200 μmol·m^−2^·s^−1^ (R:B = 9:1). The light intensity was measured at plant canopy level using a light quantum meter (LI-250A, LI-COR, USA). Plants were subjected to five different light treatments for 30 days (720 h) as shown in Fig. 1. 16 h light/8 h dark was taken as the control and recorded as L16/D8, in which there were totally 30 L/D cycles in the tested period (720 h). Similarly, 24 h light/12 h dark, 48 h light/24 h dark, 96 h light/48 h dark and 120 h light/60 h dark, which had 20, 10, 5 and 4 L/D cycles in the tested period were respectively recorded as L24/D12, L48/D24, L96/D48 and L120/D60.

### Determination of photosynthesis and chlorophyll fluorescence

The third fully expanded leaf of lettuce randomly selected from each treatment was used for photosynthesis measurement (CIRAS-3, PPSYSTEMS, USA) and chlorophyll fluorescence determination (Handy-PEA, Hansatech, UK). For photosynthesis measurements, the light, temperature, CO_2_ concentration and VPD of the leaf chamber was respectively set at 200 μmol·m^−2^·s^−1^ (R:B = 9:1), 24  °C, 400 μmol·mol^−1^ and 1.1 kPa. The O-J-I-P fluorescence induction curve obtained by JIP test analysis was used to read and calculate the photochemical efficiency and energy flow distribution parameters of PSII reaction center^24^. After dark adaptation for 20 min, the selected leaves were exposed to saturated pulsed light (3000 μmol·m^−2^·s^−1^) for 1 to 2 s. The parameters obtained were F_o_ (fluorescence at 20 μs, phase O), F_k_ (fluorescence at 300 μs, phase K), F_j_ (fluorescence at 2 ms, phase J), F_m_ (maximum fluorescence, phase P), RC (reaction center), ABS (light energy absorbed by antenna pigment), V_j_ (relative variable fluorescence of J point), M_o_ (initial slope of fluorescence induction curve of O-J-I-P) and CS (unit area). The relevant calculation formulas were as follows:

1 $$ \varphi {\text{p}}_{{\text{o}}} - {\text{the maximum photochemical efficiency of PS II under dark adaptation}}:\varphi {\text{p}}_{{\text{o}}} = \left( {{\text{F}}_{{\text{m}}} - {\text{ F}}_{{\text{o}}} } \right)/{\text{F}}_{{\text{m}}} $$

Ψ_o_—probability of the trapped exciton transferring electrons to other electron acceptors downstream of Q_A_^-^ in the electron transfer chain:

2 $$ \Psi_{{\text{o}}} = { 1 } - {\text{ V}}_{{\text{j}}} $$

3 $$ {\text{ABS}}/{\text{CS}}{-\!\!-}{\text{optical energy absorbed}}:{\text{ABS}}/{\text{CS}} \approx {\text{F}}_{{\text{o}}} $$

4 $$ {\text{TR}}_{{\text{o}}} /{\text{CS}}{-\!\!-}{\text{optical energytrapped}}:{\text{TR}}_{{\text{o}}} /{\text{CS }} = \, \varphi {\text{p}}_{{\text{o}}} \times \left( {{\text{ABS}}/{\text{CS }}} \right) $$

5 $$ {\text{ET}}_{{\text{o}}} /{\text{CS}}{-\!\!-}{\text{optical energyused for electron transfer}}:{\text{ET}}_{{\text{o}}} /{\text{CS }} = \, \Psi_{{\text{o}}} \times \left( {{\text{ TR}}_{{\text{o}}} /{\text{CS}}} \right) $$

6 $$ {\text{DI}}_{{\text{o}}} /{\text{CS}}{-\!\!-}{\text{thermal dissipationin per unit area}}:{\text{DI}}_{{\text{o}}} /{\text{CS }} = \, \left( {{\text{ ABS}}/{\text{CS}}} \right) \, - \, \left( {{\text{ TR}}_{{\text{o}}} /{\text{CS}}} \right) $$

7 $$ {\text{RC}}/{\text{CS}}{-\!\!-}{\text{the density of active reaction centers per unit area}}:{\text{RC}}/{\text{CS }} = \, \varphi {\text{p}}_{{\text{o}}} \times \left( {{\text{ V}}_{{\text{j}}} /{\text{ M}}_{{\text{o}}} } \right) \times \left( {{\text{ ABS}}/{\text{CS}}} \right) $$

8 $$ {\text{PI}}_{{{\text{abs}}}} {-\!\!-}{\text{the performance index based on absorbed optical energy}}:{\text{PI}}_{{{\text{abs}}}} = \, \left( {{\text{RC}}/{\text{ABS}}} \right) \times \left[ {\varphi {\text{p}}_{{\text{o}}} / \, \left( {{1 } - \, \varphi {\text{p}}_{{\text{o}}} } \right) \, } \right] \times \left[ { \, \Psi_{{\text{o}}} /\left( {{1 } - \, \Psi_{{\text{o}}} } \right) \, } \right] $$

### Determination of energy use efficiency

The parameters related to the energy use efficiency of lettuce planted in the plant factory were determined according to Kozai^25^. There were four parameters that were respectively the electric use efficiency (EUE), the light use efficiency (LUE), the number of photons required to produce 1 g of dry weight (*p*) and the electricity consumed to produce 1 g of dry weight (K). The calculation formulas were as follows:

9 $$ EUE = \frac{{DW \times W_{{{\text{che}}}} \times S \times D_{{}} }}{{P \times {\text{T}}}} $$

10 $$ LUE = \frac{{DW \times W_{{{\text{che}}}} \times D}}{{W_{{\text{r}}} \times {\text{T}}}} $$

11 $$ p = \frac{PPFD \times T}{{DW \times D}} $$

12 $$ K = \frac{P \times T}{{DW \times D}} $$

EUE—the electric use efficiency (%); LUE—the light use efficiency (%); *p*—the number of photons required to produce 1 g of weight (μmol·g^−1^); K—the electricity consumed to produce 1 g of weight (J·g^−1^); DW—dry weight (g); W_che_—the chemical energy corresponding to1 gram of dry weight (2 × 10^4^ J·g^−1^ ) ; S—the cultivation area (m^−2^); D—the planting density (plant·m^−2^); P—the actual power of LED panels (W); T—the cultivation time (s); W_r_—the photosynthetically active radiation received by plant canopy per unit area (W·m^-2^); PPFD— photosynthetic photon flux density (μmol·m^−2^·s^−1^).

### Determination of chlorophyll and carotenoid

A total of 0.2 g fresh samples from the mature leaves of lettuce were ground in a mortar, and then the ground was washed using 80% acetone and subsequently filtered (repeated until the leaf turned white). The filtrates were diluted to a total volume of 100 ml with distilled water. The absorbance of the extraction at 470 nm, 645 nm, and 663 nm was respectively measured by a TU-1810s spectrophotometer (PERSEE, Beijing, China). Concentrations of the chlorophyll and carotenoid were determined using the following Eqs.^26^:

13 $$ {\text{Chl a (mg/g)}} = \tfrac{{(12.72 \times {\text{OD}}663 - 2.59 \times {\text{OD}}645){{\rm V}}}}{{1000{{\rm W}}}} $$

14 $$ {\text{Chl b (mg/g) = }}\tfrac{{(22.88 \times {\text{OD}}645 - 4.67 \times {\text{OD}}663){\text{V}}}}{{1000{\text{W}}}} $$

15 $$ {\text{Car (mg/g)}} = \frac{{((1000 \times {\text{OD}}4{70} - 3.27 \times {\text{Chl.a}} - 104 \times {\text{Chl.b}})/229){\text{ V}}}}{{1000{\text{ W}}}} $$

V is the total volume of acetone extract (mL) and W is the fresh weight (g) of the sample.

### Determination of carbohydrate

Soluble sugar: 0.5 g lettuce shoot sample (DW) mixed with 10 ml distilled water was extracted in boiling water bath for 30 min (twice), after filtered, the filtrate was made to 25 ml by adding distilled water. 0.5 ml extract mixed with1.5 ml distilled water, 0.5 ml anthrone ethyl acetate reagent and 5 ml concentrated sulfuric acid was shaken and immediately put into boiling water bath and kept warm for 1 min. The extracting after natural cooling was tested at 620 nm using TU-1810s spectrophotometer (PERSEE, Beijing, China).

Starch: The mixture of 1.0 g lettuce shoot sample (DW) and 5 ml 80% (v/v) ethanol was extracted in a 80 °C water bath for 30 min. After centrifuged at 12,000×*g* for 10 min (repeated twice), the precipitate mixed with 3 ml deionized water were boiled for 15 min to gelatinise the starch. After cooling, 2 ml 30% (v/v) HClO_4_ was added and agitated, then the total volume was made to 10 ml by adding distilled water. Afterwards the solution was centrifuged at 12,000×*g* for 10 min (repeated twice) and the supernatant was collected. The glucose liberated in the supernatant was determined with the sulfuric acid anthrone method at a wavelength of 620 nm using TU-1810s spectrophotometer (PERSEE, Beijing, China)^27^.

Crude fiber: 5.0 g lettuce shoot sample (DW) was successively digested with 1.25% sulphuric acid and 1.25% sodium hydroxide, after fully dried, the residue was put in a high-temperature furnace at 550 °C for ashing. Crude fiber was estimated from the loss in weight on ignition of the dried residue using the following equation:

16 $$ Fiber({\text{\% )}} = \tfrac{{\text{loss of weight on ignition}}}{{\text{weight of sample used}}} \times {100} $$

### Determination of nitrate

The mixture of 0.5 g lettuce shoot sample (FW) and 6 mL deionized water were heated in a 80 °C water bath for 30 min and then cooled, afterwards filtered twice. Deionized water was supplemented into the filtrate to a total volume of 100 mL. Later, 0.4 mL of 5% (w/v) salicylic acid (in pure H_2_SO_4_) and 9.5 mL of 8% NaOH was added into 0.1 mL solution taken from the total 100 mL solution. Finally the nitrate content was measured at a wavelength of 410 nm with TU-1810s spectrophotometer (PERSEE, Beijing, China)^28^.

### Determination of vitamin C

The mixture of 0.2 g lettuce shoot samples (FW) and 15 mL of 4.5% aqueous phosphoric acid was shaken at 300 rpm for 30 min in the darkness, afterwards the solution was centrifuged at 16,000*g* for 10 min and the supernatants was collected. Then, the supernatants was eluted with 0.21% phosphoric acid at a flow rate of 0.8 mL/min. The vitamin C content was determined at 254 nm according to the ascorbic acid standards (Standard substance center, China) via the HPLC system (Agilent, model-1100, USA)^29^. The characteristic parameters of C18 column (Restek USA, Bellefonte, PA, USA) equipped on the HPLC system were: particle diameter—5 μm, inner diameter—4.6 mm, length—250 mm.

### Statistical analysis

Statistical analyses were conducted by one-way analysis of variance (ANOVA), using SPSS statistic software (PASW statistics version 11.0, SPSS Inc., Chicago, USA). The differences among mean values were established by Tukey's multiple range test at the 0.05 level.

### Plant material statement

The study complies with local and national regulations. No collection of seeds or plants are involved in this study.

## Results and analysis

### Plant morphology and growth dynamics of lettuce under different light/dark cycles

**Figure 2:**
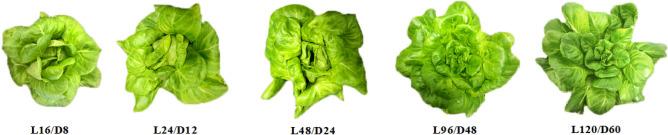
The morphology of lettuce exposed to different L/D cycle treatments of RB. L/D—light/dark. L16/D8—16 h light/8 h dark (as control), L24/D12—24 h light/12 h dark, L48/D24—48 h light/24 h dark, L96/D48—96 h light/48 h dark, L120/D60—120 h light/60 h dark. (The URL link of the software used in the figure is http://adobe.com/cn/products/photoshop.html).

As seen in Fig. 2, the morphology of lettuce differed among different treatments. The plant morphology of lettuce under L16/D8, L24/D12 and L48/D24 treatments was compact, whose leaves were closed to the plant center and the highest closure degree of leaves was detected under L48/D24. With the number of L/D cycle decreased, the leaves of lettuce exposed to L96/D48 and L120/D60 tended to be stretch and straight. It suggested that intermittent irradiation could be used to change the plant morphology through different L/D cycles without adding light photon amount or energy consumption, which has certain reference significance in the cultivation of ornamental plants.

**Figure 3:**
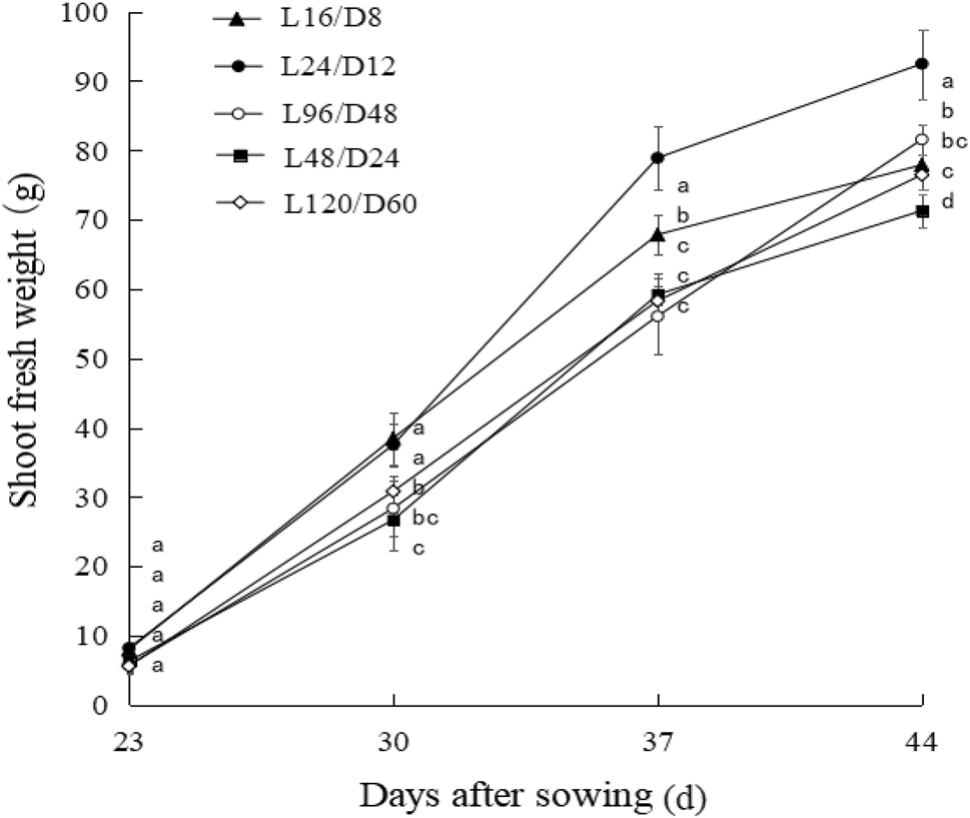
The fresh weight development of lettuce shoot exposed to different L/D cycle treatments of RB. L/D—light/dark. L16/D8—16 h light/8 h dark (as control), L24/D12—24 h light/12 h dark, L48/D24—48 h light/24 h dark, L96/D48—96 h light/48 h dark, L120/D60—120 h light/60 h dark.

As shown in Fig. 3, compared with the control, the growth rate of lettuce fresh weight under L24/D12 and L48/D24 was obviously increased especially from the 30th day and 37th day (DAS). In the whole growth period, the average growth rate of lettuce fresh weight under L24/D12 was 18.7% higher than that with the control. At harvest, the fresh weight of lettuce subjected to L24/D12 and L48/D24 was increased by respectively 18.7% and 4.7% compared with the control. It was also observed that stem diameter and plant height of lettuce exposed to L24/D12 and L48/D24 were significantly higher than the other treatments.

### LUE and EUE of lettuce under different light/dark cycles

**Figure 4:**
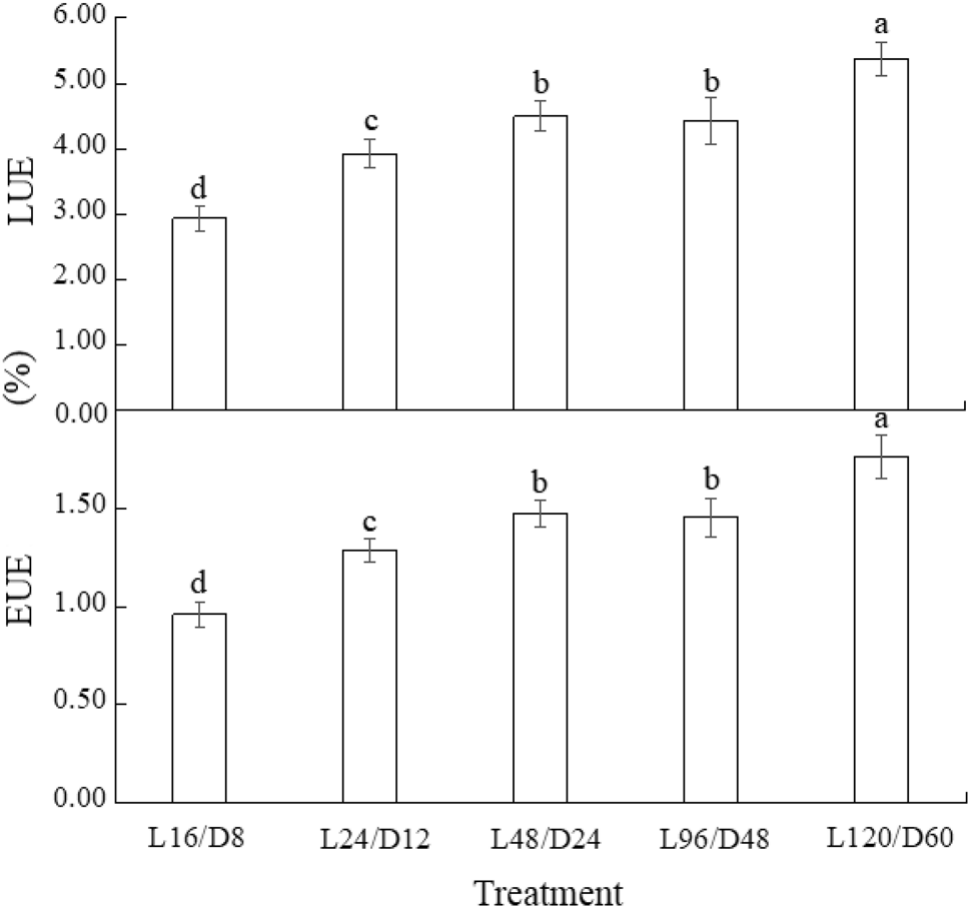
The LUE and EUE of lettuce exposed to different L/D cycle treatments of RB. Different letters for the same parameter indicate significant differences at the 5% level, according to the Tukey’s test (n = 3). The bars represent the standard errors. LUE—the light use efficiency; EUE—the electric use efficiency. L/D—light/dark. L16/D8—16 h light/8 h dark (as control), L24/D12—24 h light/12 h dark, L48/D24—48 h light/24 h dark, L96/D48—96 h light/48 h dark, L120/D60—120 h light/60 h dark.

As shown in Fig. 4, all unconventional L/D cycle treatments enhanced the LUE and EUE of lettuce. LUE and EUE of lettuce approximately increased with the length of L/D cycle extended. The highest LUE (5.37%) and EUE (1.76%) were both detected in lettuce treated with L120/D60, which were significantly higher than those with the other treatments. Although the highest fresh weight was detected in lettuce subjected to L24/D12, the LUE and EUE (calculated based on DW) of lettuce exposed to L24/D12 was significantly lower than the other abnormal L/D cycle treatments in the study.

As seen in Table 1, the least light quantum number and power consumption required to produce 1 g biomass (DW) of lettuce was respectively 1.98 mol and 1.13 MJ, which was detected under L120/D60 treatment. All the extended L/D treatments significantly increased shoot DW (by 34–83%), however, shoot FW did not show the same trend. The value of DW/FW was obviously increased with the length of L/D cycle prolonged, extended L/D period may have impacts on the absorption and utilization of water in lettuce. Additionally, root biomass (FW and DW) of lettuce under L24/L12 was significantly higher than the other treatments, the highest shoot FW and leaf thickness detected in lettuce subjected to L24/D12 may be related to the vigorous growth of root. Specific L/D cycle seemed to strengthen root growth and water absorption of lettuce.

**Table 1:** Biomass, morphological parameters, and consumption for producing per unit weight of lettuce exposed to different L/D cycle treatments of RB.

Treatment	Shoot (g)		Root (g)		Leaf number	Leaf thickness (mm)	Stem diameter (mm)	Plant height (mm)	Plant width (mm)	DW/FW (Shoot)	*P *(mol·g^−1^)	K (MJ·g^−1^)
	FW	DW	FW	DW								
L16/D8	77.89bc	2.44d	6.16b	0.35b	29c	0.31b	6.97d	102c	200c	0.031c	3.63a	2.08a
L24/D12	92.47a	3.27c	7.38a	0.43a	33bc	0.42a	9.32a	149a	230b	0.035c	2.71b	1.55b
L48/D24	81.56b	3.74b	6.16b	0.32b	38b	0.35b	8.61b	141a	240b	0.046b	2.37c	1.36c
L96/D48	71.29d	3.69b	6.05b	0.29b	37b	0.30b	7.61c	135b	275a	0.052b	2.40c	1.37c
L120/D60	76.45c	4.47a	6.28b	0.37b	42a	0.32b	7.86c	130b	290a	0.059a	1.98d	1.13d

Values for the same parameter with different letters significantly differ at the 5% level (by Tukey’s test, n = 3).

*FW* fresh weight, *DW* dry weight, *p* the number of photons required to produce 1 g of weight, *K* the electricity consumed to produce 1 g of weight.

*L/D* light/dark. L16/D8—16 h light/8 h dark, L24/D12—24 h light/12 h dark, L48/D24—48 h light/24 h dark, L96/D48—96 h light/48 h dark, L120/D60—120 h light/60 h dark.

### Pigment of lettuce under different light/dark cycles

**Figure 5:**
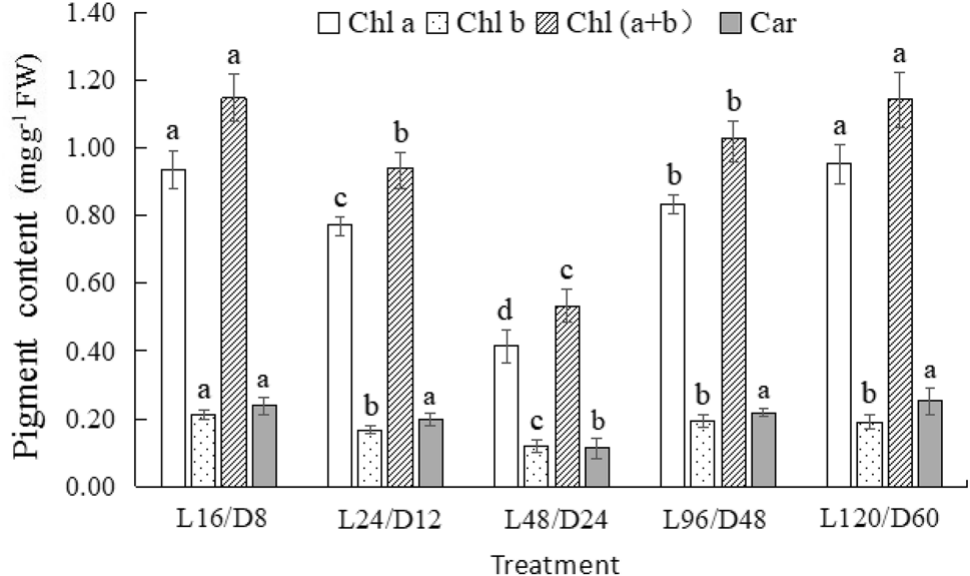
The Chlorophyll (Chl) and Carotenoid (Car) contents of lettuce exposed to different L/D cycle treatments of RB. Different letters for the same parameter indicate significant differences at the 5% level, according to the Tukey’s test (n = 3). The bars represent the standard errors. L/D—light/dark. L16/D8—16 h light/8 h dark (as control), L24/D12—24 h light/12 h dark, L48/D24—48 h light/24 h dark, L96/D48—96 h light/48 h dark, L120/D60—120 h light/60 h dark.

Figure 5 demonstrated the pigment content of lettuce subjected to different L/D cycles. The chlorophyll (Chl) and carotenoid (Car) content of lettuce descended first and then ascended among the extended L/D treatments. The minimum value of Chl and Car content was both observed in lettuce treated with L48/D24, significantly lower than those with the other treatments. The downward trend of lettuce pigments in L24/D12 and L48/D24 may be an adaptive protection mechanism for light period extension. However, with the darkness period continuously extended, the turnover of D1 protein was inhibited. Thus, the uptrend of pigment content observed in L96/D48L and 120/D60 seemed to be performance of another protection mechanism.

### Photosynthesis and chlorophyll fluorescence of lettuce under different light/dark cycles

As shown in Table 2, all abnormal L/D cycle treatments increased the assimilation rate of lettuce leaves to varied degrees compared with the control. The assimilation rate of lettuce leaves was the highest under L96/D48 and L120/D60 treatments (no significant difference between them), which was about 54.7% higher than the control. This implied that prolonging the period of L/D cycle stimulated some segments of photosynthesis thus improved the assimilation rate of leaves.

**Table 2:** Photosynthetic parameters of lettuce leaves exposed to different L/D cycle treatments of RB.

Treatment	A (μmol·m^-2^·s^-1^)	E (mmol·m^-2^·s^-1^)	Ci (μmol·mol^-1^)	gs (mmol·m^-2^·s^-1^)	WUE (%)
L16/D8	5.3c*	2.78b	431b	168bc	1.91b
L24/D12	7.7b	2.17c	398c	140c	3.55a
L48/D24	6.5bc	4.08a	554a	286a	1.59b
L96/D48	8.1a	2.51b	503a	190b	3.23a
L120/D60	8.3a	2.45b	469b	173bc	3.39a

Values for the same parameter with different letters significantly differ at the 5% level (by Tukey’s test, n = 3).

A—Assimilation rate; E—Transportation rate; Ci—Internal CO_2_; gs—Stomatal conductance; WUE—Water use efficiency.

L/D—light/dark. L16/D8—16 h light/8 h dark, L24/D12—24 h light/12 h dark, L48/D24—48 h light/24 h dark, L96/D48—96 h light/48 h dark, L120/D60—120 h light/60 h dark.

As seen in Table 3, the F_v_/F_m_ value of lettuce leaves under all treatments were greater than 0.8, indicating that abnormal L/D cycles did not result in light environmental stress for lettuce plants. V_j_ reflects the closure degree of PSII active reaction center under 2 ms illumination, while Ψ_o_ reflects the opening degree of PSII active reaction center under 3 ms illumination. The results showed that the active reaction of PSII in lettuce exposed to L24/D12 demonstrated the highest openness degree, which was significantly higher than the other treatments. RC/CS implies the number of reaction centers in PSII per unit area. The RC/CS in lettuce treated with L120/D60 was significantly higher than that with the other treatments, on the contrary, RC/CS in lettuce exposed to L96/D48 was significantly lower than any other treatment. Therefore, it was difficult to judge whether the extension of L/D cycle period was conducive to the activation of reaction centers. PI_abs_ is a photosynthetic performance index based on three main functional steps (light energy absorption, excitation energy trapping, and conversion of excitation energy to electron transport) of photosynthetic activity by a PSII reaction centre, which can reflect the state of photosynthetic apparatus^30,31^. The current results showed that PI_abs_ in lettuce subjected to L24/D12 treatment was significantly higher than the other treatments.

**Table 3:** The chlorophyll fluorescence parameters of lettuce leaves exposed to different L/D cycle treatments of RB.

Treatment	F_v_/F_m_	V_j_	Ψ_o_	RC/CS	PI_abs_
L16/D8	0.88a	0.47b	0.53b	131.48bc	2.83b
L24/D12	0.86a	0.44c	0.56a	141.21b	2.96a
L48/D24	0.87a	0.48b	0.52b	144.47b	2.83b
L96/D48	0.87a	0.52a	0.48c	111.71c	2.03c
L120/D60	0.87a	0.50a	0.50c	170.30a	2.80b

Values for the same parameter with different letters significantly differ at the 5% level (by Tukey’s test, n = 3).

F_v_/F_m_—the maximum photochemical efficiency of PSII under dark adaptation; V_j_—relative variable fluorescence of J point; Ψ_o_—probability of the trapped exciton transferring electrons to other electron acceptors downstream of QA^-^ in the electron transfer chain; RC/CS—the density of active reaction centers per unit area; PI_abs_—the performance index based on absorbed optical energy.

L/D—light/dark. L16/D8—16 h light/8 h dark, L24/D12—24 h light/12 h dark, L48/D24—48 h light/24 h dark, L96/D48—96 h light/48 h dark, L120/D60—120 h light/60 h dark.

**Figure 6:**
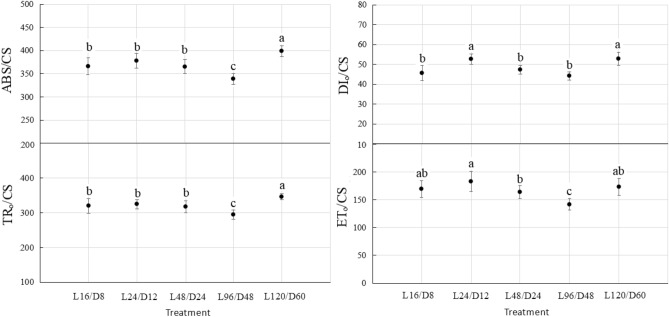
The energy flow allocations in the reaction center of PS II of lettuce exposed to different L/D cycle treatments of RB. Different letters for the same parameter indicate significant differences at the 5% level, according to the Tukey’s test (n = 3). The bars represent the standard errors. ABS/CS—optical energy absorbed; TR_o_/CS—optical energy trapped; ET_o_/CS—optical energy used for electron transfer; DI_o_/CS— thermal dissipation in per unit area. L/D—light/dark. L16/D8—16 h light/8 h dark (as control), L24/D12—24 h light/12 h dark, L48/D24—48 h light/24 h dark, L96/D48—96 h light/48 h dark, L120/D60—120 h light/60 h dark.

JIP-test was used to measure and calculate the specific activity of photosynthetic apparatus in lettuce i.e. various quantum efficiencies per unit light area, including absorption (ABS/CS), capture (TR_o_/CS), electron transfer (ET_o_/CS) and heat dissipation (DI_o_/CS). As shown in Fig. 6, the ABS/CS, TR_o_/CS and DI_o_/CS of lettuce leaves subjected to L120/D60 treatment were the highest or had no significant difference with the maximum value, indicating that prolonging the L/D cycle period might be beneficial to the absorption and capture of light energy by photosynthetic apparatus. Despite the similar ABS/CS, TR_o_/CS and significantly higher DI_o_/CS in lettuce exposed to L24/D12 relative to the control, the ET_o_/CS in lettuce treated with L24/D12 was higher than the control. The moderate extension of the L/D cycle period in L24/D12 seemed to improve the quantum efficiency of electron transfer.

### Carbohydrate contents of lettuce under different light/dark cycles

**Figure 7:**
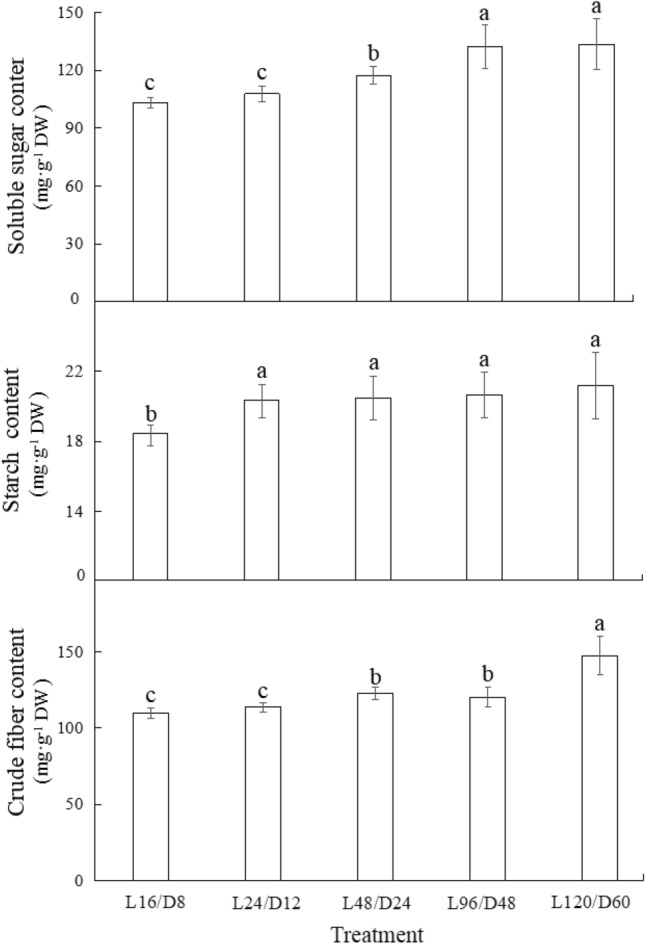
The contents of soluble sugar, starch and crude fiber of lettuce exposed to different L/D cycle treatments of RB. Different letters for the same parameter indicate significant differences at the 5% level, according to the Tukey’s test (n = 3). The bars represent the standard errors. L/D—light/dark. L16/D8—16 h light/8 h dark (as control), L24/D12—24 h light/12 h dark, L48/D24—48 h light/24 h dark, L96/D48—96 h light/48 h dark, L120/D60—120 h light/60 h dark.

The carbohydrate accumulations of lettuce exposed to different L/D cycles were displayed in Fig. 7. Compare with the control, all extended L/D cycle treatments significantly enhanced the content of starch in lettuce by 10% ~ 15%, but no significant difference was detected among the abnormal L/D cycle treatments. Except L24/D12, all treatments significantly increased the content of soluble sugar and crude fiber in lettuce compared with the control. The soluble sugar and crude fiber content showed an overall upward trend as the length of L/D cycle extended, and the highest content of soluble sugar and crude fiber were both observed in lettuce subjected to L120/D60. Lettuce is a kind of vegetable that is usually eaten raw, it is generally believed that sweetness and crispness are respectively related to the content of sugar and fiber content, and relatively high soluble sugar content and low crude fiber content are often accompanied with better taste of lettuce^32^. Therefore, as regards of lettuce taste, intermittent irradiation with relatively short L/D cycle period was better for crispness while that with long L/D cycle period was better for sweetness of lettuce.

### Vitamin C and nitrate content of lettuce under different light/dark cycles

**Figure 8:**
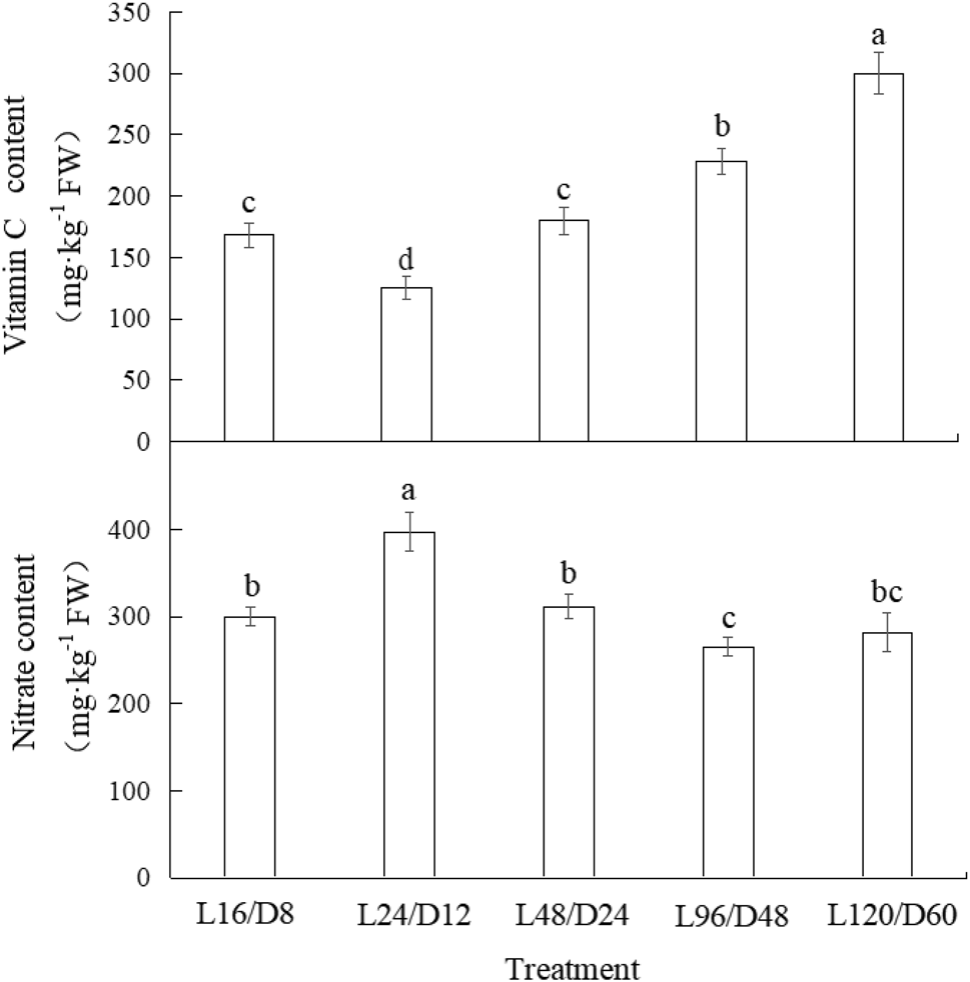
The contents of Vitamin C and nitrate of lettuce exposed to different L/D cycle treatments of RB. Different letters for the same parameter indicate significant differences at the 5% level, according to the Tukey’s test (n = 3). The bars represent the standard errors. L/D—light/dark. L16/D8—16 h light/8 h dark (as control), L24/D12—24 h light/12 h dark, L48/D24—48 h light/24 h dark, L96/D48—96 h light/48 h dark, L120/D60—120 h light/60 h dark.

As shown in Fig. 8, vitamin C content in lettuce treated with L24/L12 was decreased by 25.3% compared with the control. On the contrary, as the L/D cycle period continued to extend, vitamin C content in lettuce increased to varying degrees (7.14% ~ 78.6%) relative to the control, and the increment reached a significant level in L120/L60 treatment. As regards of the nitrate content in lettuce, L24/L12 treatment significantly increased nitrate content in lettuce by 32.2% in comparison with the control, while no significant difference was resulted as the L/D cycle period continued to extend.

## Discussion

Biological rhythm (also known as circadian clock) is a synchronous periodic change of physiological metabolism formed by biological responses to the rhythmic changes of physical factors in the natural environment (e.g. diurnal light change and seasonal temperature change). The main characteristics of circadian clock are endogeneity and inducibility. Biological physiology and behavior can still operate freely in a certain periodic rhythm even in the absence of external environmental signals and other timing factors (zeitgeber, ZT)^33,34^, which is called endogeneity. When the constant external environment changes (e.g. the L/D cycle in the current study), the endogenous mechanism of plant law is broken and the biological rhythm is reset actively to keep synchronized with the new environmental cycle^35^, which is called inducibility. Circadian rhythm system is composed of many self-sustaining cellular oscillators, which produce strong output rhythm synchronously with each other. Light and dark signal is regarded as the most powerful time signal generator, which can carry cellular oscillators and reset all oscillators to full synchronization^36^. In the present study, abnormal L/D cycles acted as timing factor (zeitgeber, ZT) in plant circadian clock system. When the L/D cycle changes regularly, the rhythmic effects of plants are actually the embodiment of the inducibility characteristics, whose performance process is as follows: plants respond to the L/D signal—regulate the biochemical reaction in the body—regulate the growth and physiological metabolism—and synchronize with the rhythmic change of light and dark signal.

Circadian clock gene is the key factor controlling the circadian clock, and the variation of L/D cycle will lead to the change of circadian clock gene expression pattern. The present results showed that different L/D cycles had great impacts on the plant morphology. Under short L/D cycles, the plant morphology of lettuce was compact and gathered, with leaves growing closed to the plant center. With the L/D cycle prolonged, plant leaves tended to be stretch and straight. The morphological differences in terms of plant height, leaf number and leaf shape of lettuce exposed to different L/D cycles may be partly regulated by Per1 gene, which is a core clock factor that plays an essential role in generating circadian rhythms and regulating cell growth and cell proliferation^37^. As reported, PIF3 (phytochrome interacting factors) can promote hypocotyl growth of plant by activating the expression of auxin synthesis genes, while the transcriptional activation activity of PIF3 is repressed by an circadian clock core component called TOC1. The inhibition is strengthened with the level of TOC1 increased during illumination, and relieved with TOC1 level decreased in darkness^38^. Thus, TOC1 may be another biological clock factor regulating lettuce morphology in the present study.

Although the circadian clock of plant shoots can affect that of roots through long-distance signals, the circadian clock gene response of different tissues is not completely consistent^39^. Li^40^ indicated that the key factors of core cellular oscillator (prr5, prr7 and prr9) regulated the activity of cell division in root meristematic zone through tzf1-tor signaling pathway, and then regulated the molecular mechanism of root morphological development. Moreover, the absorption, transport, utilization and metabolism of nitrogen in plant roots are closely related to the regulation of circadian clock, and nitrogen can also be used as a timing factor to form a negative feedback loop to reset the circadian clock^41,42^. Simon et al. (2020)^43^ compared WUE in gene mutants and overexpressed plants of Arabidopsis, showing that circadian clock had significant impacts on WUE. Although the relevant mechanisms needs to be further studied, the study by Simon et al. (2020) indicated that plant WUE can be regulated by circadian clock gene. In this study, plants exposed to L24/D12 demonstrated best root growth accompanied with the highest WUE, as well as the highest nitrate accumulation and water adsorption, which may be the results of circadian clock gene regulation. Developed roots and high water absorption may lead to a significant increase in FW of lettuce subjected to L24/D12 compared with the control, however, no significant increase of FW was observed in other extended L/D cycle treatments. That was to say, the circadian clock gene regulation results were not positively/negatively correlated with the length of the L/D cycle, reflecting the complexity of the circadian clock feedback loop. In addition, the inconsistency in circadian clock responses between shoot and root may also partly explain the different change trend of lettuce FW and DW among treatments in the present study.

All extended L/D cycle treatments significantly increased shoot DW of lettuce in comparison with the control, which could be partly explained by the increased A. It indicated that extending the L/D cycle was beneficial for the accumulation of dry matter in lettuce. However, a decline in pigment content of lettuce was detected with the L/D cycle extended, this may be an adaptive regulation to reduce light energy capture and eliminate potential photoinhibition caused by extended light period. Obviously, extended L/D period enhanced the use efficiency of pigments. Moreover, according to Matsuo et al. (2006)^44^ who demonstrated that chloroplast activities were regulated by circadian rhythm genes, the decreased pigment content observed in extended L/D treatments (especially L48/D24) may also be results of circadian clock gene regulation, although the regulation mechanism needs to be further revealed. It is worth mentioning that no significant difference was observed in photosynthetic rate of lettuce between L120/D60 and L96/D48, but significant difference was detected in shoot biomass, this may be due to the increased photosynthetic units (increased leaf number) in L120/D60.

Different from the conventional L/D cycle period (24 h) in nature to which plants are adapted, extended light period may bring about ROS (reactive oxygen species) build up or photoinhibition. However, in the present study, no obvious damage or arrested development has been detected in plants exposed to abnormal L/D cycles. Moreover, the F_v_/F_m_ value which reflects the effects of environmental stress on the photosynthetic apparatus^45,46^ was found to be similar and greater than 0.8 in all treatments of the study. As known, vitamin C is one of the important antioxidants which can clear free radicals (e.g. reactive oxygen) and protect membranes from oxidation through conversing itself to semi dehydroascorbic acid and dehydroascorbic acid. It was observed that extended light period in L96/D48 and L120/D60 resulted in significantly higher vitamin C content of lettuce. Thus, this may be an adaptive phenotype for plants to cope with the possible ROS build up. Similarly, Bie^47^ proposed that antioxidant enzymes (SS, SOD, POD, CAT) in buffalo grass demonstrated synergy with the changes of L/D cycle.

In our previous study^23^ involving shortened L/D cycle effects on lettuce, shoot biomass (both FW and DW) were significantly decreased under L6h/D3h while significantly increased by L2h/D1h relative to L16h/D8h. It indicated that either prolonged or shortened periods may result in an increase or decrease in biomass, lettuce biomass did not change unidirectionally with the extension or shortening of the L/D cycle. Some important substances in plant life activities are stimulated by light, while others are promoted in the dark. For example, proline accumulation in plant leaves was stimulated by light while suppressed in the dark, while DnaJ proteins are mainly expressed in the dark and negatively regulated by light^48–52^. Therefore, L/D cycle with appropriate intervals may result in beneficial effects in some ways by balancing the positive regulation of light and darkness. Nevertheless, the complex adaptation and synergy mechanism of lettuce to/with the L/D cycle needs to be further explored.

The cost of artificial light source and energy consumption are key factors restricting the development of PFAL. The cost of artificial light source accounts for more than 30% of the total construction cost of a PFAL, while the operating energy consumption of artificial light source accounts for more than 50% of the total energy consumption in a PFAL^53^. The results in this study demonstrated a possible way to improve the utilization rate of LED light source as well as the energy use efficiency in PFAL by extending the length of L/D cycle. In the study, based on the same total quantum number and electric power consumption in each treatment, all prolonged L/D cycle treatments were found to significantly increase the LUE and EUE of lettuce compared with the control, among which, L120/D60 resulted in the highest LUE, EUE and shoot DW of lettuce. As known, movable cultivation beds have already been applied in automated production lines in PFAL (e.g. the PFAL in Sanan Sino-Science Photobiotech Co., Ltd., China), the extended L/D cycle in this study (e.g. L120/D60) can save at least one-third of the light sources via infrequent movements of the cultivation beds, thus improving the utilization rate of LED light source and giving full play to the advantages of long life of LED chip.

## Conclusion

Different L/D cycle period affected the energy use efficiency, photosynthesis and nutritional quality of butter leaf lettuce. The DW of lettuce as well as the contents of soluble sugar and crude fiber showed an overall upward trend with the length of L/D cycle extended. Longer L/D cycle period was beneficial for the assimilation efficiency and dry matter accumulation in lettuce leaves, while specific L/D cycle seemed to strengthen root growth and water absorption of lettuce. The highest LUE, EUE, vitamin C content as well as low nitrate content were detected in lettuce treated with L120/D60, while the highest shoot FW, root biomass as well as nitrate content were observed in lettuce subjected to L24/D12. From the perspective of energy use efficiency and nutritional quality, L120/D60 was the recommended treatment in this study, while L24/D12 was the optimal treatment for FW of lettuce. No obvious stress or injury was detected in lettuce subjected to abnormal L/D cycles in terms of plant growth and production. Irradiation modes with extended L/D cycle had the potential to improve energy use efficiency and quality of lettuce in PFAL.

## Data Availability

The data used to support the findings of this study are included within the article.
